# Pilot Study of CT-Based Radiomics Model for Early Evaluation of Response to Immunotherapy in Patients With Metastatic Melanoma

**DOI:** 10.3389/fonc.2020.01524

**Published:** 2020-08-25

**Authors:** Zhi-long Wang, Li-li Mao, Zhi-guo Zhou, Lu Si, Hai-tao Zhu, Xi Chen, Mei-juan Zhou, Ying-shi Sun, Jun Guo

**Affiliations:** ^1^Key Laboratory of Carcinogenesis and Translational Research (Ministry of Education), Department of Radiology, Peking University Cancer Hospital & Institute, Beijing, China; ^2^Key Laboratory of Carcinogenesis and Translational Research (Ministry of Education), Department of Renal Cancer and Melanoma, Peking University Cancer Hospital & Institute, Beijing, China; ^3^School of Computer Science and Mathematics, University of Central Missouri, Warrensburg, MO, United States; ^4^School of Information and Communication Engineering, Xi'an Jiaotong University, Xi'an, China

**Keywords:** malignant melanoma, immunotherapy, radiomics, computed tomography, pseudoprogression

## Abstract

**Objective:** Determine the performance of a computed tomography (CT) -based radiomics model in predicting early response to immunotherapy in patients with metastatic melanoma.

**Methods:** This retrospective study examined 50 patients with metastatic melanoma who received immunotherapy treatment in our hospital with an anti-programmed cell death-1 (PD-1) agent or an inhibitor of cytotoxic T lymphocyte antigen-4 (CTLA-4). Thirty-four patients who received an anti-PD-1 agent were in the training sample and 16 patients who received a CTLA-4 inhibitor were in the validation sample. Patients with true progressive disease (PD) were in the poor response group, and those with pseudoprogression, complete response (CR), partial response (PR), or stable disease (SD) were in the good response group. CT images were examined at baseline and after the first and second cycles of treatment, and the imaging data were extracted for radiomics modeling.

**Results:** The radiomics model based on pre-treatment, post-treatment, and delta features provided the best results for predicting response to immunotherapy. Receiver operating characteristic (ROC) analysis for good response indicated an area under the curve (AUC) of 0.882 for the training group and an AUC of 0.857 for the validation group. The sensitivity, specificity, and accuracy of model were 85.70% (6/7), 66.70% (6/9), and 75% (12/16) for predicting a good response.

**Conclusion:** A CT-based radiomics model for metastatic melanoma has the potential to predict early response to immunotherapy and to identify pseudoprogression.

## Introduction

Melanoma is one of the most commonly encountered malignant tumors in the clinic and is also one of the fastest growing malignant tumors. In recent years, immunotherapies, especially those targeting the programmed cell death protein 1 (PD-1) and PD-1 ligand 1 (PD-L1) pathway, have greatly improved the treatment of melanoma. Phase 1b studies of pembrolizumab, which targets the PD-1 receptor, reported a response rate of 16.7% and a median overall survival time of 12.1 months when used as a second-line therapy for metastatic melanoma in Chinese patients (1). However, the rates of response to these anti-PD-L1 agents are lower in melanoma patients from China than patients from Western countries (2). Consequently, there is a need to better predict the responses of melanoma patients to immunotherapy.

Conventional response criteria, such as Response Evaluation Criteria in Solid Tumors (RECIST) version 1.1, might not be applicable because pseudoprogression and other patterns of atypical response occur in patients who receive immunotherapy (3, 4). When the initial evaluation is progressive disease (PD), the lack of effective methods to predict pseudoprogression may affect the confidence of doctors in continuing drug therapy. In other words, misreading of PD may lead to inappropriate treatment strategies.

Radiomics extracts a large amount of information from the CT images for quantitative analysis, data mining, and big data analytics to predict the survival and treatment efficacy of patients with different cancers. There have been major breakthroughs in the use of radiomics for lung cancer, colorectal cancer, and other tumors (5–14). To the best of our knowledge, no studies have yet applied radiomics specifically to melanoma. We hypothesize that quantification of the morphological characteristics of CT images from patients with melanoma may be useful as predictive markers. Thus, we developed and validated a radiomic model to analyze CT images of melanoma patients before and after immunotherapy to predict those who have early response to immunotherapy.

## Materials and Methods

### Patients

Data from 34 patients were used as a training sample. These patients received a PD-1 inhibitor (pembrolizumab) for treatment of metastatic malignant melanoma in our hospital from August 2016 to November 2017. Data from 16 other patients were used as a validation sample. These patients received anti-CTLA-4 immunotherapy (ipilimumab) in our hospital from January to November 2017. Inclusion criteria: All eligible patients had pathologically confirmed metastatic melanoma and received enhanced CT examination before treatment. At baseline, each patient had at least one measurable lesion based on RECIST version 1.1 criteria. After 1 and 2 cycles of immunotherapy, enhanced CT examinations were performed again to evaluate treatment response. Exclusion criteria: The contrast enhancement effect of CT in patients was not good for clinical diagnosis. Diffused invasive metastatic lesions are difficult to distinguish tumor boundaries and thus cannot be measured.

This retrospective study were reviewed and approved by our institutional review board. All patients provided written informed consent.

### CT Examination

CT scans were performed using two 64-detector row CT scanners (LightSpeed 64, GE Healthcare, Milwaukee, WI, USA and Philips Brilliant CT, Amsterdam, Netherlands). CT scans of different body parts (neck, thorax, abdomen, pelvis, and limbs) used routine scanning parameters for these regions. All patients received contrast enhanced scans, and high-pressure syringes were used to inject the elbow veins with a non-ionic iodine contrast agent at 3–4 mL/s (Ultravist, 370 mg/mL; Bayer, Germany). The dose of the contrast agent ranged from 100 to 150 mL, depending on body sites and enhanced phases.

### CT Image Analysis and Evaluation of Response to Immunotherapy

For the training sample and validation sample, the response to immunotherapy was evaluated using the RECIST version 1.1 standard. After the first cycle of anti-PD-1 therapy, the efficacy was rated as complete response (CR), partial response (PR), progressive disease (PD), or stable disease (SD). For a patient with an evaluation of PD after the first cycle, if the tumor lesion continued to increase after the second cycle of treatment, then the response was recorded as “true PD.” However, if the tumor became smaller or stabilized after the second cycle treatment, the response was recorded as “pseudoprogression.” After two cycles of anti-PD-1 immunotherapy, patients who had true PD were classified in the poor response group, and those who had pseudoprogression, PR, CR, and SD were classified in the good response group.

The two radiologists, one with 10 years and the other with 20 years experience in body CT performed image analyses jointly to agreement. They examined all CT images and identified the largest melanoma target lesion in each patient at baseline and after the first cycle of treatment. He used open-source image analysis software (3D Slicer, version 4.8.1, www.slicer.org) to manually outline the largest tumor lesions on the CT images at baseline and after the first cycle of treatment. The image of the lesion and associated data were collected for radiomics model building and prediction of the response to immunotherapy.

### Radiomics

#### Feature Extraction

In total, 497 radiomic features were extracted from the ROI region of baseline examination and after one cycle of immunotherapy treatment for each patient which included intensity, geometry, and textures features of the tumor. Delta radiomic features were computed by calculating the difference for a given feature from the two different examinations. After that we get three types of features (baseline features, one cycle of treatment features and delta radiomic features) which have seven different combinations as shown in Table 1.

**Table 1:** Image features extracted in pre-, post-and delta types.

**Intensity features**	**Texture features**	**Geometry features**
Minimum	Energy	Volume
Maximum	Entropy	Major diameter
Mean	Correlation	Minor diameter
Stand deviation	Contrast	Eccentricity
Sum	Texture variance	Elongation
Median	Sum-mean	Orientation
Skewness	Inertia	Bounding box volume
Kurtosis	Cluster shade	Perimeter
Variance	Cluster prominence	
	Homogeneity	
	Max-probability	
	Inverse variance	

##### Feature normalization

We made use of Z-scores method to normalize each feature so that the mean of each feature is 0 and the variance of each feature is 1.

##### Feature selection

Step 1: *T*-test
  This is a 2-classes classification. Features of two classes are examined by *T*-test. *P*-value (*T*-value) is used to help to select the features in step 2.
Step 2: Redundancy
  To reduce the influence of redundancy between features on prediction results, feature reduction was performed on pre-treatment features, post-treatment features, and delta-features, respectively. Calculate the cross-correlation between each two features. If the cross-correlation is larger than 0.8, the feature with larger T value in the previous step is removed.

### Radiomics Method

The training phase use multi-objective optimization to fully generates the Pareto-optimal model set. Because feature selection may impact on model parameter training, we conduct feature selection and model parameter training simultaneously. Assume that α = {α1,…, αM} is used to define the model parameters, where M is the number of model parameters. And β = {β1,…, βN} is used to define all the features, where N is the number of features. The optimization goal of the model is to maximize both sensitivity f_sen_ and specificity f_spe_ to obtain the Pareto-optimal model set, that is:

$$\begin{array}{l} \text{f}=\max_{\alpha ,\beta }\left(f_{sen},f_{spe}\right) \end{array}$$

**Figure 1:**
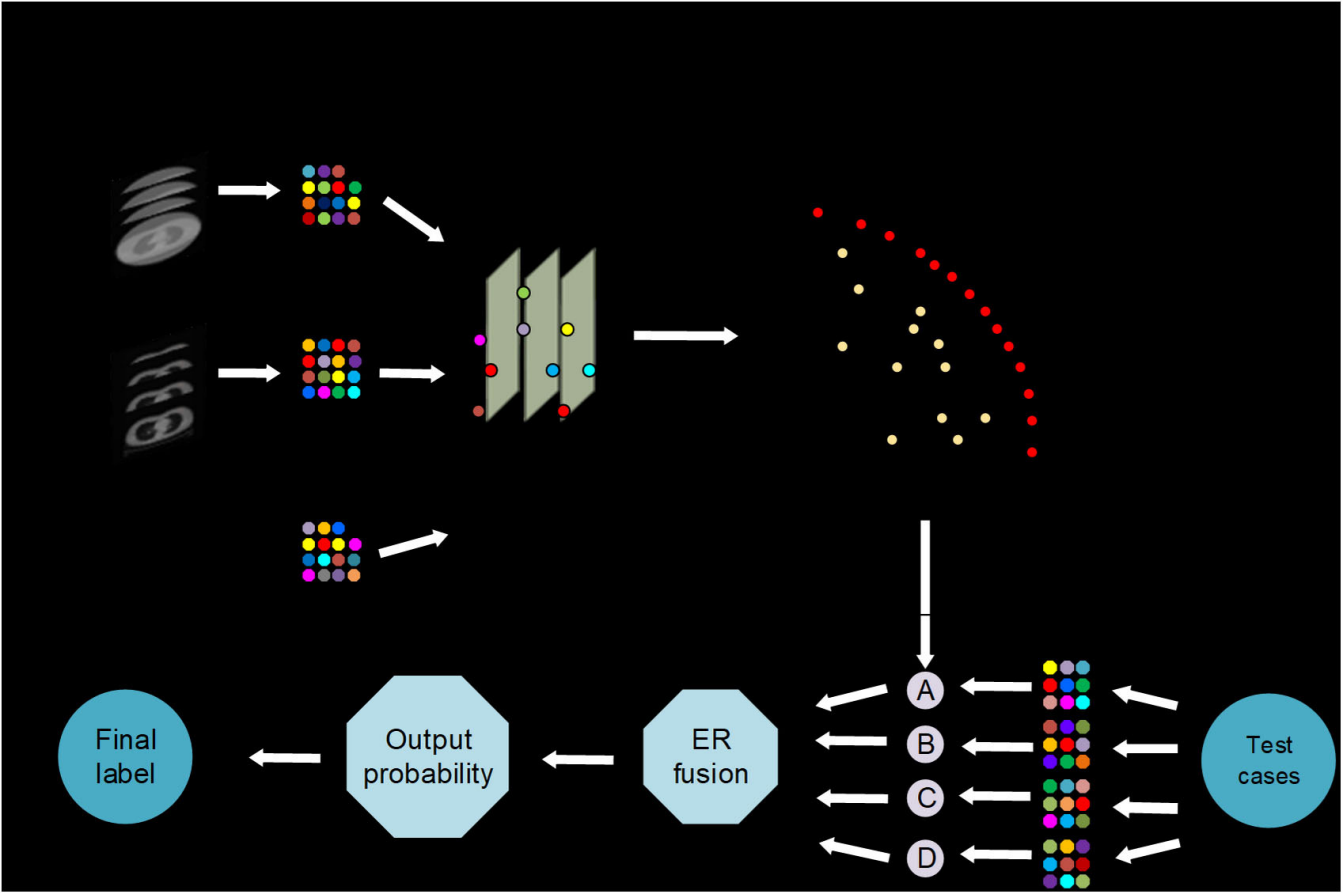
AutoMO framework, showing the workflow of the method, which has training and validation stages. It consists of training and testing stages. In training stage, a Pareto-optimal model set is generated. In testing stage, the validation samples are fed into the trained Pareto-optimal models and the final probability output is obtained through evidential reasoning strategy.

The iterative multi-objective immune algorithm (IMIA) (1) are adopted here to optimize the above objective function. IMIA includes six steps: initialization, clonal operation, mutation operation, deleting operation, updating solution set, and termination detection. In initialization, we used a hybrid initialization and initialize model parameters randomly. Clonal operation adopted the proportional cloning method (2). Mutation operation was conducted only when the mutation probability which is generated randomly is greater than the denoted mutation probability. After conducting the above two steps, there may be the same solutions in the generated solution set. So we need to perform deleting operation to keep the unique solution. AUC based the fast non-dominated sorting approach 1 is adopted to updating solution set in order to keep the solution set size. When the generation reaches the maximum number of iterations, the algorithm ends; otherwise it goes to the clone operation. The Pareto-optimal models denoted by F = {F1,…, FL} are produced after the training stage, where L is the number of Pareto-optimal models (Figure 1).

In the test phase, weight calculation, and evidential reasoning based fusion were performed (3). We used ω = {ω1,…, ωJ} to define weight, where 0 ≤ ωj ≤ 1 and $\sum_{j=1}^{J}\omega_{j}=1$. Assume that is used to define the output probability for each models, where ${\text{p}}_{j}^{1}$ and ${\text{p}}_{j}^{2}$ are the output probability for treatment response label, and ${\text{p}}_{j}^{1}+p_{j}^{2}=1$. In order to get the balanced result, the model which have non-zero weights represents a good balance between sensitivity and specificity. While the other models have zero weights. AUC is also used to compute the weights because of its ability to evaluate the model reliability. Finally, the weights are computed as:

$$\begin{array}{l} w_{j}=\left\{ \begin{array}{c} \frac{f_{sen}^{j}}{f_{spe}^{j}}+AUC_{j}\;when\;0.5\leq \frac{f_{sen}^{j}}{f_{spe}^{j}}\leq 1\\ \frac{f_{spe}^{j}}{f_{sen}^{j}}+AUC_{j}\;when\;0.5\leq \frac{f_{spe}^{j}}{f_{sen}^{j}}\leq 1\\ \;0\;Other\;situation\; \end{array}\right. \end{array}$$

where ${\text{f}}_{sen}^{j}$, ${\text{f}}_{spe}^{j}$ and *AUC*_j_ represent the sensitivity, specificity and AUC for model ι in training stage. After that, we normalize the weight. Finally, the final output probability P^*^ is got by using ER (10) to incorporate the output probabilities of different models, that is:

$$\begin{array}{l} P_{i}^{*}=\frac{\mu \times \left[\prod_{j=1}^{J}\left(\omega_{j}P_{i}^{j}+1-\omega_{j}\sum_{i=1}^{2}P_{i}^{j}\right)-\prod_{j=1}^{J}\left(1-\omega_{j}\overset{M}{\underset{i=1}{\sum }}P_{i}^{j}\right)\right]}{1-\mu \times \left[\prod_{j=1}^{N}\left(1-\omega_{j}\right)\right]},\\ \;i=1,2\;\\ \mu ={\left[\overset{2}{\underset{i=1}{\sum }}\overset{J}{\underset{j=1}{\prod }}\left(\omega_{j}P_{i}^{j}+1-\omega_{j}\overset{2}{\underset{i=1}{\sum }}P_{i}^{j}\right)-\left(J-1\right)\overset{J}{\underset{j=1}{\prod }}\left(1-\omega_{j}\overset{2}{\underset{i=1}{\sum }}P_{i}^{j}\right)\right]}^{-1} \end{array}$$

The label which has maximal output probability is the final label as:

$$\begin{array}{l} L=\text{max}({P_{i}}^{*}). \end{array}$$

### Statistical Analysis

#### Radiomics Signature Building

The prediction target is the real progress after immunotherapy, and the model was verified internally using the 5-fold cross-validation method. A support vector machine (SVM), with a radial basis function as the kernel, was used to build the model. The AUC, accuracy (ACC), sensitivity (SEN), and specificity (SPE) were used to evaluate model performance. Results were compared using an unpaired *t*-test at a significance level of 0.05.

Statistical analysis was conducted using R software (version 3.5.0; http://www.Rproject.org) and MATLAB (version 2017a; Mathworks, Natick, MA). A two-sided *P*-value below 0.05 was considered significant. MATLAB software was used to model the training sample for prediction of the good response group. The diagnostic area under the curve (AUC) of a receiver operating characteristic (ROC) curve for the training sample was obtained. Then, data from the validation group were entered into the radiomics model for calculation of the AUC.

## Results

### Patient Characteristics

We examined 50 patients with advanced melanoma, 34 in the training group and 16 in the validation group (Table 2). Patients in both groups received two cycles of immunotherapy. At that time, 18 patients in the training group had true PD, 2 had pseudoprogression, and 14 had SD or PR; 9 patients in the validation group had true PD, 3 had pseudoprogression, and 4 had SD or PR. We classified all patients as having a poor response (PD) or a good response (all other outcomes).

**Table 2:** Characteristics of patients with metastatic melanoma in this study (*n* = 50).

**Characteristic**	**Training sample *N* = 34**	**Verification sample *N* = 16**	***P*-value**
Median age (range), years	55 (31–77)	55 (47–75)	0.227
Sex			0.161
Female	22	7	
Male	12	9	
Location of primary lesion			0.466
Nasal cavity	1		
Oral cavity		1	
Esophagus	2		
Genital tract	3	1	
Rectum	1		
Skin	24	12	
Unknown	3	2	
Location of metastatic target lesion			0.299
Lung	8	3	
Lymph nodes	14	10	
Nasal cavity		1	
Subcutaneous	7	1	
Breast	1		
Adrenal gland	2		
Pleura	1		
Genital tract	1	1	

### Radiomics

**Figure 2:**
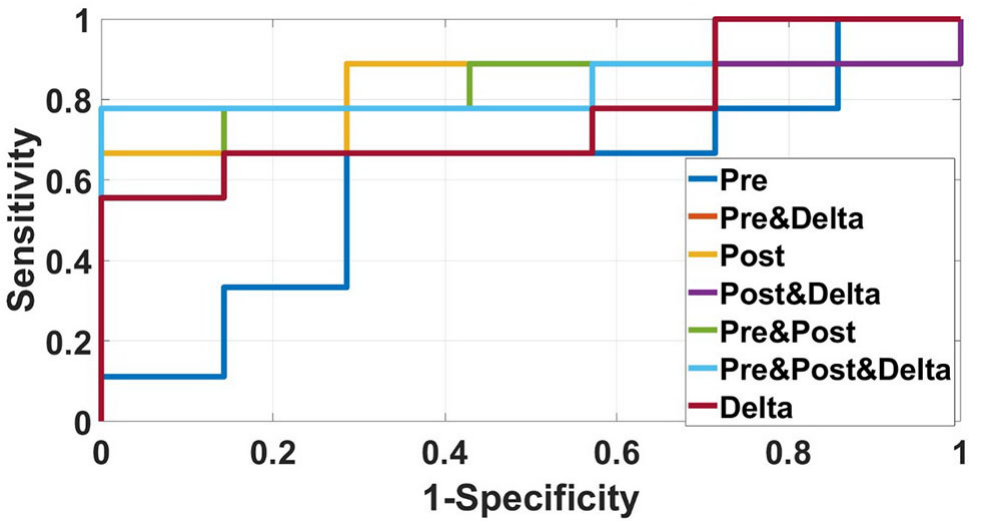
ROC curves of the radiomics model based on several CT images' features in the validation sample for predicting response to immunotherapy were showed in this figure. The best AUC (0.857) was performed by the radiomics model based on pre-treatment, post-treatment, and delta features.

We used data from patients who received anti-PD-1 immunotherapy (training group) to train the predictive model, and data from patients who received anti-CTLA-4 immunotherapy (validation group) to test the model by calculation of SEN, SPE, AUC, ACC based on different combinations of features (Table 3). The results show that pre-treatment and post-treatment delta features provided the best performance among the seven feature combinations considered. The sensitivity, specificity, and accuracy of model were 85.70% (6/7), 66.70% (6/9), and 75% (12/16) for predicting a good response. Three pseudoprogression cases were predicted as good response in the validation group (Table 3). The highest AUC of the training sample was 0.882 and the highest AUC of the validation sample was 0.857 (Table 4 and Figure 2). The number of selected features for seven combinations is shown in Table 5 and the corresponding selected features are shown in Table 6.

**Table 3:** The results of radiomics model predicting the response of immunotherapy in the validation group.

**Radiomics results**	**Clinical response results after two cycle treatments**					
	**Good response**		**Poor response**	**Sensitivity**	**Specificity**	**Accuracy**
	**Pseudoprogression**	**PR or SD**	**True progressive**			
Good response	3	3	3	85.70%	66.70%	75%
Poor response	0	1	6			

*PR, partial response; SD, stable disease*.

**Table 4:** Diagnostic performance of the radiomics model in the validation sample using different features of pre-treatment and post-treatment CT images.

**Features used**	**SEN**	**SPE**	**ACC**	**AUC**	***P*-value**
Pre-treatment	0.571	0.667	0.625	0.603	<0.0001
Pre-treatment and delta	0.857	0.667	0.750	0.810	<0.0001
Post-treatment	0.571	0.889	0.75	0.825	0.0001
Post-treatment and delta	0.714	0.778	0.750	0.778	0.0001
Pre-treatment and post-treatment	0.571	0.889	0.75	0.841	<0.0001
Pre-treatment, post-treatment, and delta	0.857	0.778	0.813	0.857	<0.0001
Delta	0.714	0.667	0.688	0.762	<0.0001

**Table 5:** Selected feature numbers for seven combinations.

	**Pre**	**Pre and delta**	**Post**	**Post and delta**	**Pre and post**	**Pre and post and delta**	**Delta**
Feature number	8	15	4	13	16	19	7

**Table 6:** Selected features for seven combinations.

	**Pre**	**Pre and delta**	**Post**	**Post and delta**	**Pre and post**	**Pre and post and delta**	**Delta**
Features	Maximum Kurtosis Major diameter Eccentricity Bounding box volume Homogeneity correlation Cluster shade	Maximum (pre) Kurtosis (pre) Bounding box volume (pre) Correlation (pre) Sum Mean (pre) Maximum (delta) Stand deviation (delta) Skewness (delta) Minor diameter (delta) Eccentricity (delta) Elongation (delta) Bounding box volume (delta) Correlation (delta) Cluster shade (delta) Homogeneity (delta)	Maximum Skewness Minor diameter Correlation	Minor diameter (post) Bounding box volume (post) Correlation (post) Homogeneity (post) Minimum (delta) Minor diameter (delta) Eccentricity (delta) Elongation (delta) Bounding box volume (delta) Sum mean (delta) Correlation (delta) Cluster shade (delta) Homogeneity (delta)	Maximum (pre) Kurtosis (pre) Major diameter (pre) Eccentricity (pre) Elongation (pre) Correlation (pre) Cluster shade (pre) Sum mean (pre) Maximum (post) Skewness (post) Minor diameter (post) Elongation (post) Bounding box volume (post) Correlation (post) Cluster shade (post) Homogeneity (post)	Maximum (pre) Kurtosis (pre) Eccentricity (pre) Bounding box volume (pre) Homogeneity (pre) Correlation (pre) Minor diameter (post) Eccentricity (post) Bounding box volume (post) Correlation (post) Cluster shade (post) Maximum (delta) Minimum (delta) Minor diameter (delta) Eccentricity (delta) Elongation (delta) Correlation (delta) Cluster shade (delta) Homogeneity (delta)	Maximum Minimum Stand deviation Skewness Minor diameter Eccentricity correlation

## Discussion

In this study, a CT-based radiomics model based on the contrast-enhanced CT images was built to predict early response to immunotherapy in patients with metastatic melanoma. The radiomics model based on pre-treatment, post-treatment, and delta features provided the best results for predicting response to immunotherapy. Receiver operating characteristic analysis for good response indicated an AUC value of 0.882 for the training group and an AUC of 0.857 for the validation group.

Cancer patients who receive immune checkpoint inhibitors experience a variety of responses, including pseudoprogression. Pseudoprogression (defined as the initial radiographic increase in tumor size or the appearance of new lesions followed by tumor shrinkage) has an incidence of about 7% in melanoma patients (15, 16). In our study, there were 5 cases (10%) of pseudoprogression among all 50 melanoma patients. In the era of immunotherapy, when the initial evaluation is progressive disease, if there is no clinical deterioration, the original treatment will continue to be used and then repeat a scan in another cycle. If the tumor is further increased after the second cycle of treatment, the treatment plan needs to be changed. If it is not confirmed, it is considered pseudoprogression and the original treatment can be continued.

Radiomics is based on a variety of imaging modalities, and numerous studies have used radiomics to evaluate the efficacy of cancer treatments. Some previous studies successfully predicted response to radiotherapy and chemotherapy. For example, Huynh et al. reported that their radiomic signature successfully predicted response to stereotactic body radiation from pre-treatment CT scans in patients with stage I/II non-small cell lung cancer (NSCLC) (17). Other studies reported a potential role for radiomics in predicting pathological response to neoadjuvant chemotherapy prior to surgery based on pretreatment CT images of NSCLC (18, 19). Liu et al. reported that their radiomics model successfully predicted pathologic CR to neoadjuvant chemoradiotherapy in patients with locally advanced rectal cancer based on pre- and post-treatment magnetic resonance imaging (MRI), and that their model could identify patients with locally advanced rectal cancer who can safely omit surgery after chemoradiotherapy (20). Cui et al. also reported that radiomics analysis of pre-chemoradiotherapy multiparameter MRI images could predict pathologic CR in patients with locally advanced rectal cancer, and their ROC analysis indicated AUCs of 0.948 (training sample) and 0.966 (validation sample) (21).

These previous studies led us to use CT imaging data to establish a radiomics model to predict the response to immunotherapy in patients with metastatic melanoma. Our model also had good AUC values in the training sample (0.882) and validation sample (0.857). Notably, our model had 100% accuracy in distinguishing pseudoprogression from poor response after the first cycle of treatment. To our best knowledge, there is currently no effective method that uses conventional CT images to identify pseudoprogression in enlarged lesions. Thus, a radiomics model that considers delta features from the images may provide more meaningful data than simple consideration of tumor size. However, we need a larger sample to confirm the ability our method to detect pseudoprogression in malignant melanoma.

The radiomic model used in the present study is an automated learning model. Compared to the multi-objective radiomics model (22), we generated Pareto-optimal models with computed weights rather than selecting an optimal model manually. We used an evidential reasoning strategy to combine the output probabilities of the non-zero weighted Pareto-optimal models to determine the final output probability. In addition, we combined traditional radiomic features and delta radiomic features to construct the predictive model. Our results demonstrated that the model performance was significantly better when combining the radiomics-delta features from before and after one cycle of treatment. This shows that consideration of changes in features during treatment has great value for predicting treatment output.

Although CTLA-4 and PD-1 works in different phase of T-cell activation, the CTLA-4 inhibitor and PD-1 inhibitor were non-specific immunotherapy, leading to a general stimulation of the immune system. Responses obtained after these immune checkpoint inhibitors are different from those observed after cytotoxic agents. Pseudoprogression, which is shown as enlargement of lesion on computed tomography (CT) imaging initially, may reflect the infiltration of T cells into tumors. Pseudoprogression could be observed in patients with advanced melanoma treated with CTLA-4 inhibitor or PD-1 inhibitor. In this preliminary study, we hope to explore whether the radiomics model can be effective in the response evaluation of the both two types of immunotherapy, so as to extend the application scope of the model to the whole immunotherapy. Meanwhile, due to the limitation of sample size, anti-PD1, and anti-CTLA4 cases were not enough to be modeled and analyzed separately, so we chose anti-PD1 cases with a larger sample size as training samples and anti-CTLA4 cases with a smaller sample size as validation samples. We also obtained the effectiveness of the model to predict the training sample itself and the ability to predict validation samples. In future studies, we hope to expand the data size and separately verify the model's ability to evaluate the efficacy of the two immunotherapy drugs.

Our study has several limitations. The region of interest of each CT image was delineated in a single slice, and therefore might not be representative of the entire metastatic lesion. Three-dimensional analysis of the entire tumor should be considered in future studies. Moreover, as a pilot study, we only examined a small-sample of patients from a single center. The power of our radiomics model needs more samples for validation. However, the results of our study provide a feasible basis for predicting pseudoprogression after immunotherapy in patients with advanced melanoma.

In summary, we developed a CT image-based radiomics model that can potentially provide early predictions of the response to immunotherapy in patients with metastatic melanoma, and identify patients with pseudoprogression. Use of this model may reduce unnecessary treatments and costs, and prevent adverse effects from chemotherapy.

## Data Availability Statement

All datasets generated for this study are included in the article/supplementary material.

## Ethics Statement

The studies involving human participants were reviewed and approved by Ethics Committee of Peking University Cancer Hospital. The patients/participants provided their written informed consent to participate in this study.

## Author Contributions

ZW, LM, LS, YS, and JG: conceptualization. ZW, LM, ZZ, and HZ: data curation. ZW, LM, ZZ, LS, and YS: formal analysis. ZW and JG: funding acquisition. ZW, ZZ, LS, HZ, XC, and MZ: methodology. ZZ, HZ, XC, and MZ: software. LS, YS, and JG: supervision. ZZ, XC, and MZ: validation. ZW and LM: writing—original draft. YS and JG: writing—review and editing. All authors: contributed to the article and approved the submitted version.

## Conflict of Interest

The authors declare that the research was conducted in the absence of any commercial or financial relationships that could be construed as a potential conflict of interest.
